# Therapeutic Drug Monitoring of Vancomycin in Hemodialysis Patients in a Hospital in North-East Romania

**DOI:** 10.3390/antibiotics14010034

**Published:** 2025-01-04

**Authors:** Aurelia Crețu, Luanda Irina Mititiuc, Iulia-Daniela Lungu, Mihaela Mihaila, Irina Dima, Adrian Covic, Cristina Mihaela Ghiciuc

**Affiliations:** 1 Department of Morpho-Functional Sciences II—Pharmacology, Clinical Pharmacology and Algeziology, Faculty of Medicine, Grigore T. Popa University of Medicine and Pharmacy of Iasi, 16 Universitatii Street, 700115 Iasi, Romania; aurelia.cretu@umfiasi.ro (A.C.); cristina.ghiciuc@umfiasi.ro (C.M.G.); 2 Pulmonary Diseases Department, “Elytis” Hospital Iasi, 43A Saulescu Street, 700010 Iasi, Romania; 3 Department of Nephrology, Faculty of Medicine, Grigore T. Popa University of Medicine, 16 Universitatii Street, 700115 Iasi, Romania; adrian.covic@umfiasi.ro; 4 Department of Nephrology, Dr. C. I. Parhon Clinical University Hospital, 700503 Iasi, Romania; lungu.iulia-daniela@d.umfiasi.ro; 5 Laboratory of Immunology, Dr. C. I. Parhon Clinical University Hospital, 50 Carol I Boulevard, 700503 Iasi, Romania; mihaela_axinte@yahoo.com; 6 Hospital Pharmacy, Dr. C. I. Parhon Clinical University Hospital, 50 Carol I Boulevard, 700503 Iasi, Romania; iridima@gmail.com; 7 Nephrology Clinic, Dialysis, and Renal Transplant Center, Dr. C. I. Parhon Clinical University Hospital, 50 Carol I Boulevard, 700503 Iasi, Romania; 8 Academy of Romanian Scientists (AOSR), 3 Ilfov Street, Sector 5, 50044 Bucharest, Romania; 9 Saint Mary Emergency Children Hospital, 700309 Iasi, Romania

**Keywords:** vancomycin, area under the curve, hemodialysis patients, critically ill patients, central venous catheter infection

## Abstract

**Background/Objectives**: Vancomycin is a reserve antibiotic that is frequently prescribed for central venous catheter (CVC)-associated infections in hemodialysis patients. Hemodialysis patients are very fragile patients and the presence of CVCs increases the risk of sepsis. We conducted a prospective study, evaluating the needs of changes in vancomycin dosing for treatment based on the use of the new 2020 vancomycin dosing guidelines, to increase drug safety (preventing subtherapeutic or supratherapeutic doses and offering therapeutic concentrations of the drug) in a particular group of patients with sepsis caused by catheter infections and being on intermittent hemodialysis. **Methods**: This prospective study included patients with sepsis caused by catheter infections and being on intermittent hemodialysis, treated with vancomycin, admitted in the nephrology department and intensive care unit (ICU). Vancomycin levels were adjusted according to the 2020 vancomycin guidelines. **Results**: In our study, nine (45%) patients had a vancomycin AUC between 400 and 600 mcg × h/mL, five (25%) patients had a subtherapeutic AUC, and six (30%) patients had a supratherapeutic AUC. It is important to mention that in 10 (50%) of the patients included in the study, the loading and maintenance doses mentioned in the protocol were respected, but 50% of them had a supratherapeutic AUC. We observed that a supratherapeutic AUC occurred when the loading dose was 1500 mg or 2000 mg, and in one case at 1000 mg with a low BMI. **Conclusions**: a therapeutic level of vancomycin can often be difficult to achieve because of different reasons, mainly in hemodialysis patients.

## 1. Introduction

Patients on hemodialysis, who have catheter infections, including sepsis, are often empirically prescribed reserve antibiotics, particularly vancomycin, for infections caused by Gram-positive bacteria that are resistant to several antibiotics [1]. Patients who receive vancomycin treatment can develop renal impairment, particularly if the recommended dosages are not adequate, used concurrently with other drugs with nephrotoxic potential, or if renal perfusion is low. A consecutive increase in serum creatinine of 0.5 mg/dL or an at-least 50% increase from the baseline measured on treatment days is considered vancomycin-induced acute kidney damage [2]. Numerous factors impact renal function in critically ill patients, necessitating a change in vancomycin dosage [3]. Patients in the intensive care unit (ICU) require every precaution to properly guide their treatment in order for therapy to be successful. Hemodialysis patients are fragile patients, a particular group in which kidney function is already lost, and administering vancomycin during the last 60–90 min of hemodialysis seems to be a recommended option to prevent vascular damage and preserve patients’ vascular access [4]. The guidelines on the administration of vancomycin in these specific patients are currently under development; the recommendations have not yet been uniformly implemented across all healthcare facilities, and all these measures are aimed at preventing toxicity, treatment failure, and reducing the emergence of vancomycin-resistant strains of the pathogens responsible for the infection, which often lead to sepsis [5].

The new 2020 vancomycin dosing guidelines signify a substantial paradigm shift in the management of intravenous vancomycin administration dosing in methicillin-resistant *Staphylococcus aureus* (MRSA) infections and other infections that can be cured with vancomycin treatment, such as *Streptococcus* spp., *Enterococcus* spp., and *Corynebacterium* spp. infections. Vancomycin can be given as a continuous infusion in patients with severe or deep-seated infections (e.g., pneumonia, endocarditis, bone and joint infections). For MRSA pneumonia, osteomyelitis, endocarditis, and bacteremia, target levels based on Ctrough are 15–20 mg/L to improve penetration to target sites [6]. The transition from trough-based dosing to area under the curve (AUC)-based dosing of vancomycin with an AUC/MICBMD ratio of 400–600 mg/hour/L (assuming a broth microdilution MIC of 1 mg/L) is mandatory to achieve clinical efficacy and ensure patients’ safety. These recommendations, with achieving AUC doses early in therapy (24 h), are necessary for the accuracy and safety of patient treatment, especially in patients with renal replacement therapy, who are critically ill, or are obese [6,7]. Historically, AUC estimation required multiple blood samples’ collection over a dosing interval, but vancomycin monitoring based on the new 2020 vancomycin dosing guidelines demands very few bloods samples’ collection and provides accurate therapy, as reviewed by other authors [6,8].

According to published data, the average frequency of these infections ranges from 0.6 to 6.5 episodes per 1000 catheter-days. *Staphylococcus aureus*, *Enterococcus*, *Klebsiella pneumoniae*, and coagulase-negative staphylococci are the primary causes of catheter infections [7]. *Staphylococcus aureus*-induced sepsis frequently leads to joint and cardiac impairment and requires long-term antibiotic treatment. In the 24 h after the implantation of any vascular catheter, bacteria can multiply in biofilm and then disseminate into the blood. Consequently, this increases the risk of hospitalization due to infections (0.34 events per person/year), as well as mortality (10% of patients died within 30 days after discharge) [9].

We conducted a prospective study, evaluating the needs of changes in vancomycin dosing in treatment based on the use of the new 2020 vancomycin dosing guidelines, to increase drug safety (preventing subtherapeutic or supratherapeutic doses and offering therapeutic concentrations of drug) in a particular group of patients with sepsis caused by catheter infections and being on intermittent hemodialysis, admitted in a hospital in north-east Romania. We have chosen a particular category of patients, hemodialysis patients on a central venous catheter, in whom inappropriate concentrations of vancomycin frequently lead to toxicity, vascular damage, or increased antibiotic resistance, as they are a category susceptible to infection and sepsis, and are high consumers of antibiotics, especially reserve antibiotics [10].

## 2. Results

### 2.1. Patients’ Characteristics

**Figure 1:**
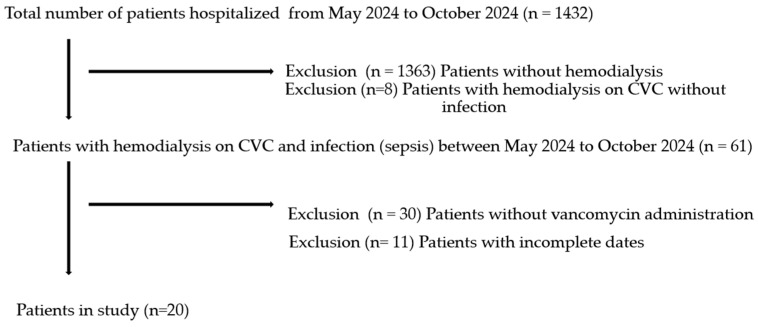
Patients’ selection.

Our study included 20 acute or chronic kidney disease patients with an intermittent hemodialysis indication on CVCs, who were undergoing vancomycin treatment in an ICU for sepsis due to CVC infection (Figure 1). From a total of 1432 patients’ hospitalizations in a study period from 20 May 2024 to 20 October 2024, 69 patients were on hemodialysis with a CVC and 61 patients developed a CVC infection, with etiologic agents depicted in Table 1*. Klebsiella*, followed by *Staphylococcus,* were the most frequent bacteria involved in etiology of sepsis.

Out of 61 patients with CVC infection, only 20 patients accepted AUC-monitored vancomycin treatment (Figure 1).

Patients’ characteristics are presented in Table 2. A total of 15 (75%) were male and 5 (25%) were female, with the mean age 56 ± 15 years. The mean body mass index (BMI) was 24.9 ± 5.7 kg/m^2^; the proportion of patients with a BMI ≥ 25 kg/m^2^ was 40%. The median SOFA score was 3 points. The main comorbidities were cardiovascular: 13 (65%) patients had arterial hypertension and 12 (60%) patients had heart failure, followed by other comorbidities such as diabetes mellitus, present in 4 patients (20%).

From all included patients (Table 3), two (10%) were infected with MRSA; from six (30%), *Staphylococcus aureus* was isolated; three (15%) had vancomycin-sensitive *Enterococcus* spp., of which two patients had *Enterococcus faecalis* sepsis; one patient (5%) had *Staphylococcus hominis* MDR; one (5%) patient had Coagulase-negative staphylococci MRSCN; in two (10%) cases, the bacteria causing sepsis were not identified; and in three (15%) cases, Gram-negative bacteria were identified, and treatment with vancomycin was stopped and changed according to the antibiogram results.

### 2.2. Treatment Characteristics

Concomitant drug analysis found the use of loop diuretics in seven cases (35%) and that of spironolactone in four cases (20%), while noradrenaline was used in four cases (20%). Concomitant antibiotic administrations with vancomycin treatment are depicted in Table 4.

After bacterial identification and use of an antibiogram in four cases, the therapy was de-escalat to oxacillin (beta-lactamases against methicillin-susceptible *Staphylococcus aureus* (MSSA)).

The average days of hospitalization in the ICU was 14 ± 8 days and the average treatment duration with vancomycin was 6 ± 4 days. We observed that four (20%) patients died, of which two patients had sepsis with MDR *Enterococcus faecalis*, one had MRSA, and one had *Pseudomonas aeruginosa*. In the four patients who died, vancomycin empiric treatment was confirmed to be efficient by antibiogram in three cases of vancomycin-susceptible bacteria, but in one case, a bacterium not sensitive to vancomycin was identified. The AUC of vancomycin was subtherapeutic in two cases (one with a normal BMI and one who was overweight), and the AUC was supratherapeutic in one case (one with a normal BMI, but near-overweight). Moreover, these patients developed a second infection: endocarditis, bronchopneumonia, or had multiple previous catheter blood stream infections.

### 2.3. AUC and Relationship Between Changes in Treatment and AUC Monitoring

In our study, nine (45%) patients had a vancomycin AUC between 400 and 600, five (25%) patients had a subtherapeutic AUC, and six (30%) patients had a supratherapeutic AUC. In one case with a supratherapeutic dose, even if the dose was reduced, new determinations revealed an increased AUC, and in another case, from a subtherapeutic dose, the patient arrived at a supratherapeutic dose.

It is important to mention that in 10 (50%) of the patients included in the study, the loading and maintenance doses mentioned in the protocol were respected, but 50% of them had supratherapeutic AUC doses: the loading doses were lower than in the protocol, while the maintenance doses were higher. We observed that a supratherapeutic AUC occurred when the loading dose was 2000 mg in obese patients (#3 and #7) or 1500 mg in overweight patients (#9 and #14; patient #1 was near-overweight and had a supratherapeutic AUC) or 1000 mg in the non-overweight patient #6 (Table 5).

## 3. Discussion

In our study, among 69 patients with intermittent hemodialysis and on a CVC, 61 presented catheter sepsis, but only 20 patients were treated under the monitoring of vancomycin and were included in our study to determine vancomycin concentrations based on AUC calculation and adjusted treatment. We observed a high incidence of CVC infections in hemodialysis patients, especially of sepsis; from 69 hospitalized hemodialysis patients in our study period, 61 had sepsis. In many cases, the etiologic agent was not specified, and *Klebsiella* spp. was identified first, followed by *Staphylococcus aureus* and *Enterococcus spp* during the studied period. The incidence of infections is higher in patients dialyzed on CVC compared to those with arteriovenous grafts or fistulas [10].

All *Enterococcus* spp. strains showed multidrug resistance (aminopenicillins, quinolones) and sensitivity to vancomycin.

Vancomycin is one reserve antibiotic that it is frequently empirically prescribed and we observed that its dose in our patients remained challenging. In literature reports on hemodialysis patients, it is noted that they receive intravenous antibiotics more than once a year in an outpatient dialysis setting and that vancomycin is the most prescribed antibiotic; sometimes, in more than 68% of cases, it is chosen as the first antibiotic to be administered intravenously. The recommendation of vancomycin in hemodialysis patients, depending on the need and the center where it is performed, seemed to be inappropriate in 37% of cases with infection after the results of cultures became accessible, and regarding surgical prophylaxis, the recommendation of vancomycin seems to be the most inappropriate [10].

In our study, 10 (50%) of the patients received the loading and the maintenance doses according to the protocol, but only four patients had adequate doses of vancomycin, with the AUC between 400 and 600, in a situation in which there were requirements to achieve clinical efficacy and ensure safety for patients. Five patients had supratherapeutic AUC doses, while one patient had subtherapeutic doses. Most of the other subtherapeutic doses were the result of non-adherence to the protocol with low loading doses administration.

Similar to a Liu Y et al. study from 2022, our patients did not obtain optimal vancomycin doses, and also the management of dose adjustment was inappropriate. In the Liu study, from 69 patients on continuous renal replacement therapy that received vancomycin and therapeutic drug monitoring, less than 15% of patients achieved the optimal concentration. Fewer patients in the subtherapeutic group received a daily dose adjustment than those in the exceeding target group [11]. In our study, 30% achieved the optimal concentration and fewer patients received an adjustment of the dose. In contrast to the mentioned study, with the exception of one case, we observed that in patients with obesity and an increased adjusted loading dose, a supratherapeutic AUC was obtained.

In two patients with a supratherapeutic AUC and subsequent Ctrough determinations after concentration adjustment, therapeutically adequate doses have not yet been obtained, and vancomycin did not reach a stable trough concentration after the third or fourth dose.

In other studies, in patients without hemodialysis vancomycin, a therapeutic level was not achieved in 16.8% of the patients [12]; in the hospital with a therapeutic drug monitoring protocol, most of the vancomycin concentrations (55.56%) were in the therapeutic range of 10–20 mg/L [13]. In critically ill patients, Bakke et al. observed that in less than 40% of the patients, there were obtained therapeutic trough serum concentrations of vancomycin during the first 3 days of treatment and patients with increased renal clearance had lower serum trough concentrations but received increased maintenance doses and frequent loading doses [14].

We observe that a supratherapeutic AUC occurred when the loading dose was 1500 mg or 2000 mg, and in one case at 1000 mg with a low BMI. In a study of patients with severe renal impairment (creatinine clearance < 30 mL/min), without hemodialysis and a loading dose of 25–30-mg/kg of vancomycin, nephrotoxicity occurred in 7.2% of patients in the high-dose group [15].

The revised guidelines now recommend AUC-guided dosing and monitoring over the trough-only approach, and important caveats must be respected. Dose must be adapted to body weight, fluid, and the AUC. Guidelines recommend a narrow predialysis serum concentration range of 15–20 mg/L, and in this mode, it is more possible to achieve therapeutic success with an AUC/MIC between 400 and 600. The time when the dose is determined is also important; many dialysis centers administer vancomycin during dialysis and doses may appear as subtherapeutic [6].

Few prospective studies have been undertaken in dialysis patients based on dose adjustment according to the AUC of vancomycin, on the one hand because of the dialysis itself and also because of the timing of blood sampling and vancomycin dosing. It makes a difference whether vancomycin is given during or after the dialysis session, because during the session, some of the vancomycin is washed away before reaching the tissues. Also, the best time to collect blood and determine the appropriate dose has also not been determined and established for therapeutic drug monitoring, to achieve the best results to increase drug safety and for patient safety [16].

We have seen how changing current practices is challenging, and recent studies bring up barriers such as cumulative experience over time, a lack of financial support to implement protocols and perform the necessary dosing, and long turnaround times for serum levels of vancomycin [17], but most patients should receive a particular intervention according to updated guidelines [18].

Studies in the literature reveal an increased number of patients in whom hemodialysis initiation is on a central venous catheter compared to an arteriovenous graft or fistula, as well as complications given by the disease and procedure, and increased morbidity and mortality. Multiple infections are associated with the use of hemodialysis on CVCs, even from the first days, and in the first year of use. CVC-related bloodstream infections are the second leading cause of mortality in patients undergoing hemodialysis following cardiovascular disease [19,20,21,22]. In a study by Kazakova in patients with permanent renal replacement, out of 1870 patients in whom hemodialysis on a CVC was initiated, in the first year of follow-up, 37.5% died, and 29.3% developed a bloodstream infection a median of 88 days after hemodialysis initiation (30–180 days) [19]. In study of Weldetensae et al. published in 2023, 418 catheters in 395 patients were placed, of which 331 were temporary and 22 were permanent, with 200 having a duration on the catheter of less than 30 days and 153 of more than 30 days, and 158 episodes of catheter-associated infections occurred in 135 patients, of which 127 were in patients with a temporary catheter [20].

In another study from 707 patients, 197 had catheter-related bloodstream infections in a 20-month study [21]. In some hemodialysis patients’ cases of infection, the researchers aimed for 62.2% of patients to have already had a previous blood stream infection before the study period [22]. Hence the major importance of the right use of antibiotics as this population is prone to infections, has an increased consumption of back-up antimicrobial chemotherapeutics as well as being antibiotic resistant.

In our study, we included only inpatients with continuous hospitalization (altered status, unstable, signs of infection), and patients in whom CVC use was initiated a number of days prior to the current hospitalization, and we did not include the patients who received hemodialysis on the day of hospitalization.

### Limitations

The study included a relatively small number of patients and offers the experience of a single center.

## 4. Materials and Methods

### 4.1. Study Design and Participants

This prospective study included patients with sepsis caused by catheter infections and being on intermittent hemodialysis, treated with vancomycin, admitted in the nephrology department and intensive care unit (ICU), in the Dr. C.I. Parhon Clinical University Hospital in Iasi, Romania. This is a regional hospital, the biggest in north-east Romania, with 70 beds in the Nephrology Clinical Department, 20 beds in the Internal Medicine Department, 25 beds in the Geriatrics and Gerontology Clinical Department, and 75 beds in the Urology and Renal Transplant Clinical Department.

The inclusion criteria were as follows: (1) acute or chronic kidney disease with an intermittent hemodialysis indication and on a central venous catheter (CVC), (2) sepsis caused by catheter infections, (3) age ≥ 18 years, and (4) completion of initial and follow-up vancomycin dose evaluations. These patients were selected from patients with acute or chronic kidney disease hospitalized between 20 May 2024 and 20 October 2024. The selected diagnosis codes were as follows: N18.0—end-stage renal disease, N18.90—unspecified chronic renal failure, N18.8—other chronic renal failure, and N17.0—acute renal disease.

The exclusion criteria were as follows: (1) age < 18 years, (2) patients on continuous hemodialysis, (3) patients on peritoneal dialysis, (4) patients with a renal transplant, (5) patients who have had a prophylactic administration of vancomycin after surgery, (6) patients requiring vancomycin treatment for *Clostridium difficile* (oral vancomycin can be used to treat *C. difficile* disease, but the oral formulation is not an effective treatment for systemic infection), (7) patients for whom vancomycin therapy has been discontinued within up to 48 h of initiation, (8) an allergy to vancomycin or other glycopeptides, and (9) missing data.

### 4.2. Vancomycin Dosing and Pharmacodynamics Data

For serum vancomycin dose determinations, we used the following: Architect IA Vancomycin Reagent Tests, an Architect IA Vancomycin Calibrator Kit, and Architect IA Wash Buffer for the Vancomycin Architect Analyzer (Abbott, Wiesbaden, Germany). At each stage of blood sampling, 2 mL of whole blood was taken by experienced nurses for laboratory analysis. For the Cpeak concentration, the serum concentration was obtained 3 h after the completion of intravenous vancomycin dosing and for Ctrough concentration, the serum was obtained 30 min before the next dose of vancomycin. Pre-hemodialysis or 3 h post-hemodialysis, the target value is 10–15 mcg/mL; Ctrough levels should be maintained >10 mg/L to avoid resistance.

To avoid ototoxicity or red man syndrome, or to prevent vascular damage, vancomycin was always administered as follows:

- By intravenous perfusion, either in 0.9% sodium chloride or 5% glucose;
- In a final concentration: no more than 5 mg/mL for peripheral administration;
- With an infusion rate not faster than 10 mg/min.

Demographic data and sequential organ failure assessment (SOFA) scores were collected from medical records at the initial vancomycin dose. Results on the etiologic agent and its sensitivity to antibiotics were also collected. The definitions of sepsis and septic shock followed “The Third International Consensus Definitions for Sepsis and Septic Shock” [23].

According to 2020 vancomycin guidelines [6], dose initiation occurred according to an initial loading dose of vancomycin, calculated on the basis of the patient’s actual body weight:

- For a weight < 40 kg, a dose of 750 mg of vancomycin is recommended in a volume of 250 mL saline solution at 0.9%, with an infusion time of 1.5 h (90 min).
- For a weight of 40–59 kg, a dose of 1000 mg of vancomycin is recommended in a volume of 250 mL saline solution at 0.9%, with an infusion time of 2 h (120 min).
- For a weight of 60–90 kg, a dose of 1500 mg of vancomycin is recommended in a volume of 500 mL saline solution at 0.9%, with an infusion time of 3 h (180 min).
- For a weight > 90 kg, a dose of 2000 mg of vancomycin is recommended in a volume of 500 mL saline solution at 0.9%, with an infusion time of 4 h (240 min).

Recommended doses are designed to achieve Ctrough of 10–15 mg/L of vancomycin; the dose is rounded up to the nearest multiple of 250 mg; a loading dose of 2 g/dose should not be exceeded; for severe infections, the loading dose may be 25–30 mg/kg.

The maintenance dose for vancomycin (vancomycin pulsed infusion—initial maintenance dosage) is undertaken according to creatinine clearance (in our cases, <15 mL/min) and patient weight—between 10 and 15 mg of the vancomycin/kg×1 dose, rounded to the nearest 250 mg, with a max dose of 1500 mg after each hemodialysis session.

If the patient has recently received vancomycin, we have revised the previous regimen and patient information to determine the appropriate current regimen. We have considered rounding the nomogram dose up or down based on patient-specific factors that have a significant impact on vancomycin distribution (e.g., pregnancy, severe trauma, ascites, high fluid intake, etc.). In patients with decompensated cirrhosis, we have reduced the daily dose by 30%. The first maintenance dose was administered at least 24 h after the loading infusion, taking into account the dosing interval established. Doses of up to 2000 mg can be diluted in 500 mL of fluid.

### 4.3. Outcomes

The main purpose of this study was to determine the proportion of patients with vancomycin AUC levels within the recommended range of 400–600 mcg·h/mL, after the application of the 2020 vancomycin dosing protocol for a judicious use of vancomycin based on two instances determining the plasma concentrations of vancomycin (Cpeak, Ctrough).

Secondary objectives focused on clinical outcomes: the length of hospital stay, treatment duration with vancomycin, the analysis of the etiologic agent that caused sepsis, adverse effects, antibiotic resistance, and mortality.

### 4.4. Statistical Analysis

Demographic data were presented as a means ± standard deviation (SD) for continuous numerical variables and as a percentage for categorical or dichotomous variables and analyzed using Microsoft Excel 365 — for Windows [24].

## 5. Conclusions

Hemodialysis patients are a special and fragile category of patients, susceptible to infections, and are high consumers of antibiotics, especially reserve antibiotics. It is important to mention that half of the patients with doses administered according to the protocol presented a supratherapeutic AUC. For a therapeutic success, to decrease antibiotic resistance and the costs associated with the care of these patients, adapted protocols with precise measurements of low therapeutic spectrum antibiotics, such as vancomycin, are necessary. The standardization of doses according to protocols based on the AUC in hemodialysis centers represents a necessity over doctors’ preferences and experience because the recommended doses often do not provide therapeutic targets. Our study represents an opportunity and may lead to the prioritization of funds to implement a vancomycin stewardship protocol administration guided by the AUC. Patients with catheter sepsis and hemodialysis are a particular group with their own particularities, disease complexity, and comorbidities, who need special attention when applying this protocol; therefore, we need to enroll more patients to establish the right doses for these patients.

## Figures and Tables

**Table 1:** Bacterial etiology of infection in hemodialysis on CVC patients (N = 61).

Bacterial Strains	n (%)
*Staphylococcus* spp.	7 (11.5%)
*Klebsiella* spp.	9 (14.8%)
*Proteus* spp.	2 (3.3%)
*Escherichia coli*	3 (4.9%)
*Acinetobacter* spp.	1 (1.6%)
Unspecified bacteria	39 (63.9%)

**Table 2:** Patients’ characteristics (N = 20).

Characteristics (N = 20)	n
Gender	
Female, n (%)	5 (25%)
Male, n (%)	15 (75%)
Age (years, mean ± SD; range)	56 ± 15; 22–83
Weight (kg, mean ± SD; range)	71 ± 18; 45–107
BMI kg/m^2^ (mean ± SD; range)	24.9 ± 5.7; 17.1–34.9
SOFA score (mean ± SD)	2.3 ± 0.9
Comorbidities	
Arterial hypertension	13 (65%)
Heart failure	12 (60%)
Diabetes mellitus	4 (20%)

BMI, body mass index; SOFA, sequential organ failure assessment.

**Table 3:** Bacterial etiology of sepsis in study patients.

Bacterial Etiology of Sepsis	n (%)
*Staphylococcus aureus*	6 (30%)
MRSA	2
Coagulase-negative staphylococci	1 (5%)
MRSCN	1
*Enterococcus faecalis*	2 (10%)
MDR	2
*Staphylococcus hominis*	1 (5%)
MDR	1
*Enterococcus* spp.	1 (5%)
MDR	1
Unidentified bacteria	2 (10%)
Negative	4 (20%)
*Morganella morganii*	1 (5%)
*Pseudomonas aeruginosa*	1 (5%)
*Klebsiella pneumoniae*	1 (5%)

MRSA, methicillin-resistant *Staphylococcus aureus*; MRSCN, methicillin-resistant coagulase-negative staphylococci; MDR, multidrug-resistant bacteria.

**Table 4:** Antibiotics associated with vancomycin treatment in study patients.

Antibiotics Associated with Vancomycin Treatment	Patients Number
Quinolones	3
Aminoglycosides	3
Cephalosporins	2
Carbapenems	2
Sulfonamide	1
Ureidopenicilline	1
Nitroimidazole	2
Without another antibiotic	3

**Table 5:** Patient demographics and serum vancomycin concentrations at different sampling times.

Patient’s Code	Demographic Data: Gender; Age (year); BMI (kg/m^2^)	Comorbidities	SOFA	Vancomycin Concentration Type	mcg/mL	Vancomycin Doses			AUC (mcg·h/mL)	AUC2 (mcg·h/mL)
						Loading Dose (mg)	Maintenance Dose (mg)	Dose Adjustment		
1	Female; 64; 24.8	HTA, HF, DM	2	C Peak	41.89	1500 mg	1000 mg	from 1000 mg to 750 mg	764.00	-
				C Trough	25.69					
2	Female; 59; 24.2		3	C Peak	27.47	1000 mg *	1000 mg *		464.00	-
				C Trough	13.54					
3	Male; 51; 32.8		3	C Peak	38.05	2000 mg	1000 mg		763.00	
				C Trough	30.58					
				C Peak 2	47.12			from 1000 mg to 250 mg; de-escalation to oxacillin		949
				C Trough 2	37.45					
4	Male; 52; 15.9	HTA, HF	3	C Peak	35.47	1000 mg	1000 mg	de-escalation to oxacillin	554.00	-
				C Trough	21.15					
5	Male; 66; 19.1	HTA	3	C Peak	18.41	1000 mg	750 mg		381.00	-
				C Trough	12.36					
6	Male; 22; 20	HTA, HF	3	C Peak	36.46	1000 mg	1000 mg *	de-escalation to ciprofloxacin	724.00	-
				C Trough	24.64					
7	Male; 55; 34.8	HTA, HF, DM	1	C Peak	39.07	2000 mg	1500 mg	from 1500 mg to 500 mg	676.00	-
				C Trough	20.32					
8	Male; 58; 20.3	HTA, HF, DM	3	C Peak	22.91	1500 mg	1000 mg *		396.00	
				C Trough	18.05					
				C Peak 2	32.14			from 1000 mg to 500 mg		674
				C Trough 2	20.89					
9	Male; 46; 28.1	HTA	3	C Peak	42.02	1500 mg	1000 mg	de-escalation to oxacillin	712.00	-
				C Trough	14.98					
10	Male; 32; 23	HTA, HF, DM	1	C Peak	36.83	1500 mg	1000 mg		442.00	-
				C Trough	14.77					
11	Male; 78; 25.4	HTA, HF	3	C Peak	22.57	1000 mg *	1000 mg	from 1000 mg to 1500 mg	333.00	-
				C Trough	11.15					
12	Male; 56; 34.9		3	C Peak	22.60	1000 mg *	1000 mg *		384.00	-
				C Trough	10.36					
13	Male; 59; 31	HTA, HF	3	C Peak	28.78	1500 mg	1000 mg		545.00	-
				C Trough	17.48					
14	Male; 46; 28.1	HTA	2	C Peak	41.98	1500 mg	1000 mg	from 1000 mg to 750 mg	666.00	-
				C Trough	20.29			de-escalation to oxacillin		
15	Female; 46; 22		2	C Peak	18.21	1000 mg *	1000 mg *		322.00	-
				C Trough	12.06					
16	Male; 83; 22	HTA, HF	3	C Peak	29.80	1500 mg	1000 mg *		524.00	-
				C Trough	19.89					
17	Male; 41; 17.1	HF	1	C Peak	21.92	1000 mg	750 mg *		430.00	-
				C Trough	16.32					
18	Female; 66; 27.7		1	C Peak	26.37	1000 mg	1000 mg		481.00	-
				C Trough	17.67					
19	Female; 75; 25.7	HF	2	C Peak	28.41	1000 mg *	1000 mg		537.00	-
				C Trough	19.70					
20	Male; 56; 17.7	HTA, HF	1	C Peak	26.30	1000 mg	1000 mg *	changed to colistin	474.00	-
				C Trough	16.60					

HTA: Hypertension; HF: heart failure; DM: diabetes mellitus; * vancomycin protocol was not respected by prescribing doctors.

## Data Availability

The original contributions presented in this study are included in the article. Further inquiries can be directed to the corresponding author.

## References

[1] Sahli F., Feidjel R., Laalaoui R. (2017). Hemodialysis catheter-related infection: Rates, risk factors and pathogens. J. Infect. Public Health..

[2] Bamgbola O. (2016). Review of vancomycin-induced renal toxicity: An update. Ther. Adv. Endocrinol. Metab..

[3] Ibe Y., Ishigo T., Fujii S., Takahashi S., Fukudo M., Sato H. (2023). Simulation of Vancomycin Exposure Using Trough and Peak Levels Achieves the Target Area under the Steady-State Concentration-Time Curve in ICU Patients. Antibiotics.

[4] El Nekidy W.S., Cha R., Ghazi I.M. (2022). Practical considerations for vancomycin dosing in hemodialysis patients: Perspectives from the nephrology stewardship pharmacist. Clin. Nephrol..

[5] Lewis S.J., Nolin T.D. (2021). New Vancomycin Dosing Guidelines for Hemodialysis Patients: Rationale, Caveats, and Limitations. Kidney360.

[6] Rybak M.J., Le J., Lodise T.P., Levine D.P., Bradley J.S., Liu C., A Mueller B., Pai M.P., Wong-Beringer A., Rotschafer J.C. (2020). Therapeutic Monitoring of Vancomycin for Serious Methicillin-resistant Staphylococcus aureus Infections: A Revised Consensus Guideline and Review by the American Society of Health-system Pharmacists, the Infectious Diseases Society of America, the Pediatric Infectious Diseases Society, and the Society of Infectious Diseases Pharmacists. Clin. Infect. Dis..

[7] Jantarathaneewat K., Phodha T., Singhasenee K., Katawethiwong P., Suwantarat N., Camins B., Wongphan T., Rutjanawech S., Apisarnthanarak A. (2023). Impact of Pharmacist-Led Multidisciplinary Team to Attain Targeted Vancomycin Area under the Curved Monitoring in a Tertiary Care Center in Thailand. Antibiotics.

[8] Pai M.P., Neely M., Rodvold K.A., Lodise T.P. (2014). Innovative approaches to optimizing the delivery of vancomycin in individual patients. Adv. Drug Deliv. Rev..

[9] Bello A.K., Okpechi I.G., Osman M.A., Cho Y., Htay H., Jha V., Wainstein M., Johnson D.W. (2022). Epidemiology of haemodialysis outcomes. Nat. Rev. Nephrol..

[10] Apata I.W., Kabbani S., Neu A.M., Kear T.M., D’agata E.M., Levenson D.J., Kliger A.S., Hicks L.A., Patel P.R. (2021). Opportunities to Improve Antibiotic Prescribing in Outpatient Hemodialysis Facilities: A Report From the American Society of Nephrology and Centers for Disease Control and Prevention Antibiotic Stewardship White Paper Writing Group. Am. J. Kidney Dis..

[11] Liu Y., Jiang L., Lou R., Wang M., Si Q. (2022). Vancomycin therapeutic drug monitoring in patients on continuous renal replacement therapy: A retrospective study. J. Int. Med. Res..

[12] Al-Maqbali J.S., Shukri Z.A., Sabahi N.A., Al-Riyami I., Al Alawi A.M. (2022). Vancomycin therapeutic drug monitoring (TDM) and its association with clinical outcomes: A retrospective cohort. J. Infect. Public Health.

[13] Mauliņa I., Darbiniece K., Miķelsone-Jansone L., Erts R., Bandere D., Krūmiņa A. (2022). Experience of Vancomycin Therapeutic Drug Monitoring in Two Multidisciplinary Hospitals in Latvia. Medicina.

[14] Bakke V., Sporsem H., Von der Lippe E., Nordøy I., Lao Y., Nyrerød H.C., Sandvik L., Hårvig K.R., Bugge J.F., Helset E. (2017). Vancomycin levels are frequently subtherapeutic in critically ill patients: A prospective observational study. Acta Anaesthesiol. Scand..

[15] Marvin J.L., Levine B.J., Papas M., Rosini J.M. (2019). An evaluation of the incidence of nephrotoxicity after a loading dose of vancomycin in patients with severe renal impairment. J. Emerg. Med..

[16] Lewis S.J., Mueller B.A. (2021). Evaluation and Development of Vancomycin Dosing Schemes to Meet New AUC/MIC Targets in Intermittent Hemodialysis Using Monte Carlo Simulation Techniques. J. Clin. Pharmacol..

[17] Alghanem S.S., Albassam A., Al-Rashidi N., Bin Haidar Z. (2023). Awareness, perception, and barriers of healthcare providers toward the revised consensus guideline for therapeutic monitoring of vancomycin. Saudi Pharm. J..

[18] Khwaja A. (2012). KDIGO clinical practice guidelines for acute kidney injury. Nephron Clin. Pract..

[19] Kazakova S.V., Baggs J., Apata I.W., Yi S.H., Jernigan J.A., Nguyen D., Patel P.R. (2020). Vascular Access and Risk of Bloodstream Infection Among Older Incident Hemodialysis Patients. Kidney Med..

[20] Weldetensae M.K., Weledegebriel M.G., Nigusse A.T., Berhe E., Gebrearegay H. (2023). Catheter-Related Blood Stream Infections and Associated Factors Among Hemodialysis Patients in a Tertiary Care Hospital. Infect. Drug Resist..

[21] Pasilan R.M., Tomacruz-Amante I.D., Dimacali C.T. (2024). The epidemiology and microbiology of central venous catheter related bloodstream infections among hemodialysis patients in the Philippines: A retrospective cohort study. BMC Nephrol..

[22] AbuTaha S.A., Al-Kharraz T., Belkebir S., Abu Taha A., Zyoud S.H. (2022). Patterns of microbial resistance in bloodstream infections of hemodialysis patients: A cross-sectional study from Palestine. Sci. Rep..

[23] Singer M., Deutschman C.S., Seymour C.W., Shankar-Hari M., Annane D., Bauer M., Bellomo R., Bernard G.R., Chiche J.-D., Coopersmith C.M. (2016). The Third International Consensus Definitions for Sepsis and Septic Shock (Sepsis-3). JAMA.

[24] Divisi D., Di Leonardo G., Zaccagna G., Crisci R. (2017). Basic statistics with Microsoft Excel: A review. J. Thorac. Dis..

